# An optimization technique for identifying robot manipulator parameters under uncertainty

**DOI:** 10.1186/s40064-016-3417-5

**Published:** 2016-10-12

**Authors:** Kuan-Lin Li, Wu-Te Yang, Kuei-Yuan Chan, Pei-Chun Lin

**Affiliations:** Department of Mechanical Engineering, National Taiwan University, Taipei, Taiwan

**Keywords:** Robot manipulator, Uncertainties, Geometric tolerance, Optimization, Trajectory planning

## Abstract

Robot manipulators enable large-scale factory automation of simple and repeated tasks. Each manipulation is the result of the robot design and the command inputs provided by the operator. In this study, we focus on the accuracy improvement of practical robot manipulation under uncertainty, resulting in path-specific error values. Existing techniques for reducing the errors use high-precision sensors and measurements to obtain the values of a manipulator to provide feedback control. Instead of compensating errors in operation, this study designs a calibration table to obtain the error value for a designated path. This error is then used to adjust important parameters in the kinematic closed chain models of a manipulators via optimization. The proposed method reduces the cost and the dependence on the calibration process. Experimental results show that the overall accuracy of the manipulator is improved. The proposed method can also be extended to develop the optimal robotic manipulation planning and reliability assessment in the future.

## Background

Robot manipulators can perform simple and repeated tasks, such as pick and place (Wallen 2008), to enable highly intelligent and highly accurate operations (Smith et al. 2012). An increasing number of companies are using robot manipulators to support the manufacturing process. With the rapid development of automation technology, there is a great demand for the high-precision robot manipulators. However, since robots are a complex systems, there are several uncertainty factors that affect the positioning error of a manipulator.

Robot accuracy is affected by several factors, such as the environmental, parameters, measurement, computational, and application (Karan and Vukobratovic 1994). These factors can be classified as kinematic, structural, or dynamics errors (Conrad et al. 2000), and geometric or non-geometric factors (Caenen and Angue 1990; Jang et al. 2001), such as link deformation (Hsueh 2012), assembly error, joint clearance (Lai 2014), and gear backlash and wear (Mukras et al. 2010). Therefore, there are 14 performance criteria (Table 1) defined in ISO 9283, which describes methods and environmental conditions for testing the accuracy, repeatability, and performance of robots.

Existing methods and standards for identifying the accuracy of robots have practical limit. In practice, some manufacturers only present one or two performance criteria, such as path repeatability and pose repeatability, omitting robot accuracy. Therefore, the robot information found in a product manual is incomplete and unable to use in comparisons (Slamani et al. 2012). Althought the testing space of an industrial serial robot in ISO 9283 could be the main operation space, robot manufactures do not state how they calculate their repeatability (Mousavi et al. 2015). Due to the effects of various uncertainties, the performance criteria changes when a robot’s workspace is out of the testing space from the manufacturer (Hsueh 2012). That is, robot accuracy and whether the workspace is out of testing space used by the manufacturer are unknown.

**Table 1:** ISO industrial robots performance criteria (ISO 1998)

Performance criteria	
Pose accuracy and pose repeatability	Path accuracy and path repeatability
Multi-directional pose accuracy variation	Path accuracy on reorientation
Distance accuracy and distance repeatability	Cornering deviations
Position stabilization time	Path velocity characteristics
Position overshoot	Minimum posing time
Drift of pose characteristics	Static compliance
Exchangeability	Weaving deviations

Although the true accuracy of a robot may be unknown, most robots are calibrated before usage. Calibration methods can be divided into two categories. In the first category (Roth et al. 1987; Mooring et al. 1991), there are two calibration levels. In Level 1, the joint sensor signal is calibrated to match the actual joint displacement. In Level 2, the kinematic model is calibrated. Specifically, the Denavit-Hartenberg (DH) (Denavit 1955) model is calibrated. Non-geometric calibration mainly deals with non-geometric factors that influence robot accuracy. The second category (Elatta et al. 2004) divides calibration methods into kinematic-model-based and non-kinematic calibrations. Generally, a calibration process entails four steps: modeling, measurement, identification, and compensation (Karan and Vukobratovic 1994).

- *Step 1: Modeling* Among the many methods used to describe the kinematics of a robotic system, the DH model is commonly used. DH parameters are modeled and error terms for each parameter are considered.
- *Step 2: Measurement* The accuracy and resolution of measurement devices influence the accuracy of robotic systems. Camera systems, laser trackers, and vision systems are commonly used for measurement.
- *Step 3: Identification* In this step, the goal is to find the error terms modeled in the first step. Linear least squares and nonlinear least squares methods are frequently applied for this purpose.
- *Step 4: Compensation* In this step, the error terms found in the previous step are added to the original kinematic (DH) model to improve the accuracy of robot arms.

**Fig. 1:**
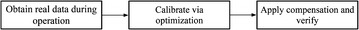
The steps in this paper

Instead of calibrating for a specific task, the present study proposes obtaining robotic parameters via a calibration table. We design the calibration table and a dual-arm system to improve the accuracy of a manipulator using an optimization method to adjust DH parameters. The proposed procedure has three steps in Fig. 1. First, real data are obtained during operation via a Vicon camera system, the calibration table, and encoders. These data are the robot’s real positions and joint angles obtained at the calibration points. Then, the summation of errors between all ideal positions and real positions is calculated. Second, calibration is conducted via optimization. An optimization framework that uses deviations of DH parameters as the design variables is then formulated to minimize the position errors. Third, the compensation is applied and verified. The robot arm is controlled to move to the verification points, which are different from the calibration points, with optimized DH parameters. Then, the position of real points is captured using the Vicon camera system and the errors are determined by comparing ideal and real positions. Finally, the accuracy improvement is assessed.

The rest of this paper is organized as follows. “The modeling and experiment setup of a 3-DOF serial robot” section describes the model and system of the robot arm. “Proposed research method for accuracy improvement and DH parameter calibration” section describes the proposed method. “Results” presents the results of verification. Finally, “Conclusions” summarizes the main results and contributions of this paper.

## The modeling and experiment setup of a 3-DOF serial robot

The robot arm used in this paper is a 3-DOF (degrees of freedom) serial robot with three revolute joints (Chou 2014). We design the calibration table and use Vicon camera system to obtain real data during operation. The Vicon camera system is also used to capture the end-effector position during verification. To assess the accuracy of a robot arm, the kinematic model of the robot is needed. In this research, the DH transformation matrix method, which is widely used to describe the kinematics of robots, is applied to construct the model of the robot system. In addition, there are many uncertainties in the actual robot. Therefore, in order to model uncertainties, a deviation matrix for DH parameters is constructed.

### Description of robot system

#### System overview

The robot system consists of two PUMA-style arms since they are commonly used in production lines and their workspaces are similar to those of human arms. Each arm has three DOFs. The left and right arms have symmetrical structures. For the context of this research, we use the right arm for testing, calibration, and the final measurement of the results. The left arm is prepared for the next stage research to form a closed-loop chain for additional measurement in the future. Therefore within the contexts of this paper, the left arm can temporary be omitted. The motor for the third DOF is installed close to the arm base to decrease inertia. Its torque is transmitted to the joint by a pulley-and-belt system. The motors of the three joints are brushed DC motors because of the requirement of high torque.

**Fig. 2:**
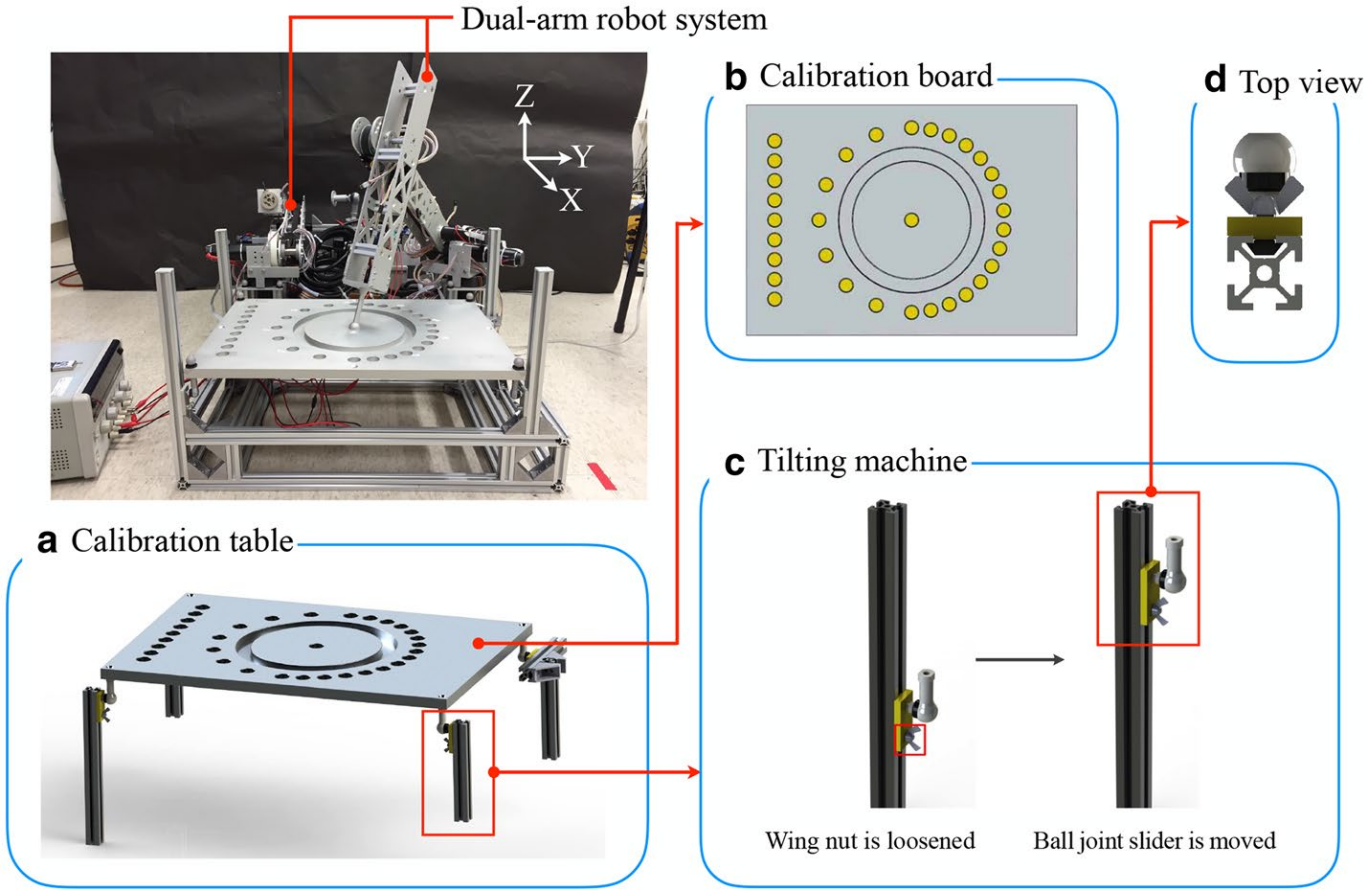
Photograph of robot arm system. **a** Photographs of calibration table. **b** Top-view photograph of calibration board. **c** Diagrams of tilting mechanism’s ball joint slider movement. **d** Top-view photograph of tilting mechanism

#### Accuracy measurement

The T-20S motion capture system (Vicon Motion Systems Ltd.) was used to measure the accuracy of our system. This system uses passive optical motion capture technology. Based on our experiments, the resolution of the system has a standard deviation of $\pm 0.28$ mm with a 95 % confidence interval. Reflective markers are tracked by cameras to get their positions. For our robot-arm system, several markers were placed on the base and end-effector so that their positions could be tracked by the cameras. The location and orientation of the calibration table relative to the robot arm can also be determined using the motion capture system.

#### Calibration table

The calibration table is shown in Fig. 2a. It comprises the calibration board, tilting mechanisms, and aluminum extrusion. The calibration board can be tilted in roll and pitch directions via the tilting mechanisms. Our proposed method to calibrate DH parameters is to form a close-loop robot using a calibration table. Through the known position of the calibration table, we could back-calculating the actual location/position of the robot in our experiment. In addition, we investigate the result of calibration on different trajectories via a tilting mechanism in the calibration table with 3 degree-of-freedom. Through the adjustment of the tilting angle, the calibration board on the calibration table can rotate in pitch and roll direction, creating calibration trajectories in different spaces.

- *Calibration board* Figure 2b shows a top-view photograph of the calibration board. There are 33 calibration points on the board, which has a cylindrical cavity. These calibration points form a circular path and a straight path.
- *Tilting mechanism* The purpose of the tilting mechanism is to increase the number of DOFs of the calibration board. It allows calibration trajectories to be in three-dimensional space. Figure 2c, d shows the tilting mechanism. To increase the number of DOFs while retaining sufficient stiffness of the calibration table, a 3-DOF ball joint with high stiffness was used in the tilting mechanism. The calibration board can tilt in roll and pitch directions. A wing nut on the ball joint slider is used to fix the ball joint slider and aluminum extrusion (see Fig. 2c, d). Therefore, the calibration broad rotates in roll and pitch direction via the tilting mechanism.

### Modeling of the robot system

#### Frame of the robot arm

**Fig. 3:**
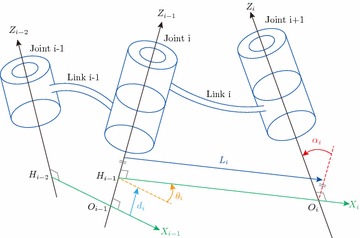
Definition of robot arm’s parameters

Frames were attached to each joint to describe its position and geometric relationship with neighbors. For a link of the robot arm, one end of it is the *i* joint and the other is the $i+1$ joint (Fig. 3). The rules used to define frames are as follows:

1. $z_{i-1}$ should coincide with the *i* axis. Either direction is allowable.
2. $x_{i-1}$ should be perpendicular to $z_{i-1}$ and $z_{i}$. When $i-1$ and *i* are parallel, the origin location for $i-1$ is arbitrary.
3. Define $y_i$ using the right-hand rule.

#### Uncertainty model of 3-DOF serial robot arm

**Fig. 4:**
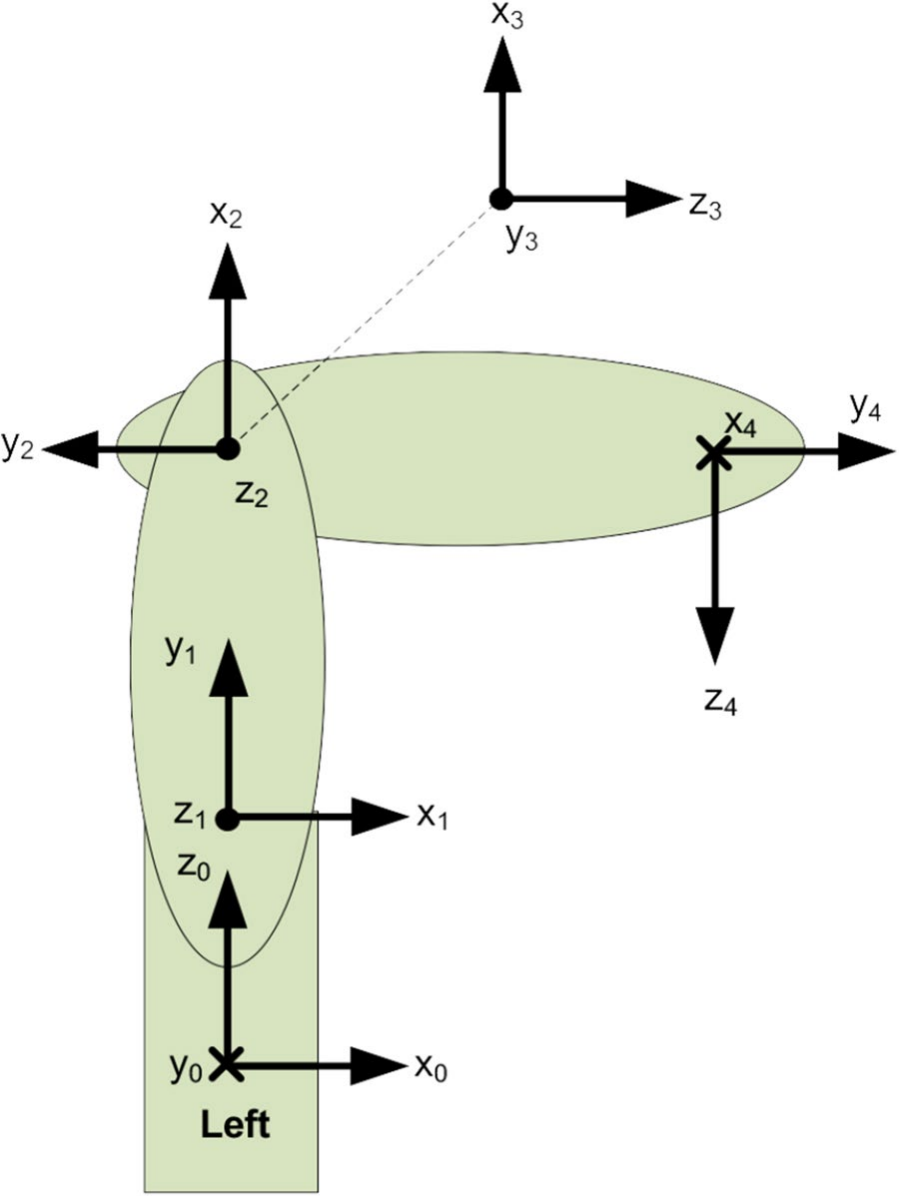
Robot model

This research focuses on 3-DOF serial robot arm. Its ideal DH parameters are shown in Fig. 4 and Table 2.

**Table 2:** Ideal DH parameters

Joint	$d_i$ (m)	$\theta _i$	$L_i$ (m)	$\alpha _i$
1	0.076	$\theta _1$	0	90°
2	0	90° + $\theta _2$	0.4	0°
3	−0.012	$\theta _3$	0	90°
4	0.3543	0°	0	90°

An ideal robot arm can be described as Eq. (1), where *f* is the robot system, *d*, $\theta$, *L*, and $\alpha$ are the DH parameters of the robot arm, and *Y* is its position information.

1 $$f(d,\theta ,L,\alpha )=Y$$

In reality, the robot arm is described as Eq. (2), where $\Delta d$, $\Delta \theta$, $\Delta L$, and $\Delta \alpha$ are its real parameters, and $\Delta Y$ is its real position information.

2 $$\begin{aligned} f(\Delta d,\Delta \theta ,\Delta L,\Delta \alpha )=\Delta Y \end{aligned}$$

Due to uncertainty factors, the DH parameters of the robot arm usually differ from the real ones, which leads to positioning errors of the robot. The uncertainty factors of each parameter are discussed below:

- *d*, *L*: *d* and *L* are affected by the geometric error of each link and result in variations $\delta d$ and $\delta L$, and thus real $d_i$ and $L_i$ are represented as $d_i+\delta d_i$ and $L_i+\delta L_i$, respectively.
- $\theta$, $\alpha$: The machining and assembly errors of joints cause variations $\delta \theta$ and $\delta \alpha$, and thus $\theta _i+\delta \theta _i$ and $\alpha _i+\delta \alpha _i$ are the real $\theta _i$ and $\alpha _i$, respectively.

The uncertainties in the DH parameters are summarized in Table  3.

**Table 3:** DH parameters with uncertainties

Joint	$d_i$ (m)	$\theta _i$	$L_i$ (m)	$\alpha _i$
1	(0.076 + $\delta d_1$)	$\delta \theta _1$ + $\theta _1$	(0 + $\delta L_1$)	90° + $\delta \alpha _1$
2	(0 + $\delta d_2$)	90° + $\delta \theta _2$ + $\theta _2$	(0.4 + $\delta L_2$)	0° + $\delta \alpha _2$
3	(−0.012 + $\delta d_3$)	$\delta \theta _3$ + $\theta _3$	(0 + $\delta L_3$)	90° + $\delta \alpha _3$
4	(0.3543 + $\delta d_4$)	0°	(0 + $\delta L_4$)	90°

### Robot control and trajectory planning

#### Robot control

**Fig. 5:**
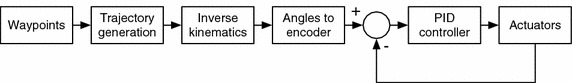
Block diagram of robot control

The robot was on position control mode and the block diagram is showed in the Fig. 5. User input waypoints into robot system and a trajectory is generated in Cartesian space. Then, angle information is obtained by inverse kinematics. Then, the angle information is changed to encoder position and serves as setpoint for robot arm. Therefore, robot arm can be controlled to move and compensated by PID controller.

#### Trajectory planning

User input waypoints into robot arm and a trajectory is generated. The trajectory is planned by cubic spline method in Cartesian space. Four condition, initial and final positions and initial and final velocities, are given to solve this function. Initial and final position are the *i*th and $(i+1)$th waypoints. Initial velocity is the desired velocity of *i*th section and final one is the desired velocity of $(i+1)$th section. The trajectory function is Eq.  (3).

3 $$\begin{aligned} s(t)=a_0+a_1t+a_2t^2+a_3t^3 \end{aligned}$$

### Comparison of actual model and ideal model

**Fig. 6:**
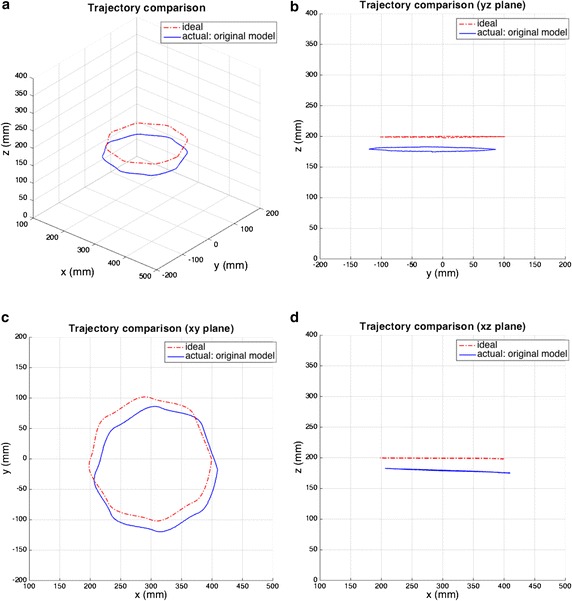
First comparison of ideal and actual trajectories. **a** Trajectory error and views of **b** yz, **c** xy, and **d** xz planes

**Fig. 7:**
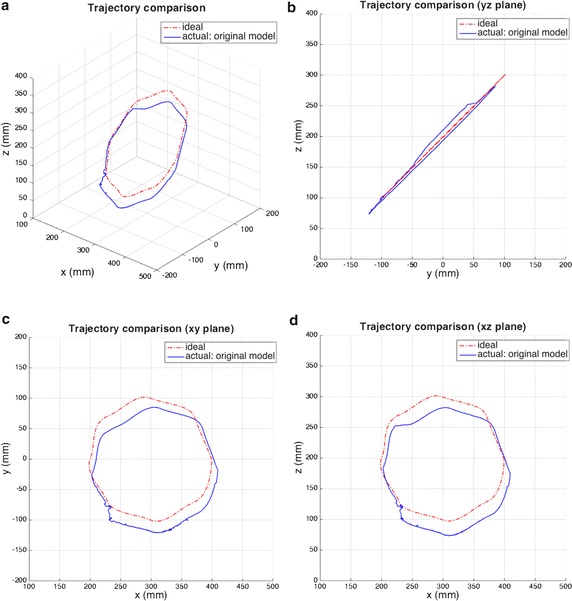
Second comparison of ideal and actual trajectories. **a** Trajectory error and views of **b** yz, **c** xy, and **d** xz planes

There are many uncertainties in an actual robot, such as those due to geometric tolerance, joint clearance, and backlash. We used various trajectories to compare the robot’s ideal model and actual model. The results are shown in Figs. 6 and 7. These figures show that the actual robot has a trajectory error of almost 3 cm and that the trend of error varies with trajectory. Consequently, we discuss an optimization technique for adjusting the DH parameters for various trajectories.

## Proposed research method for accuracy improvement and DH parameter calibration

**Fig. 8:**
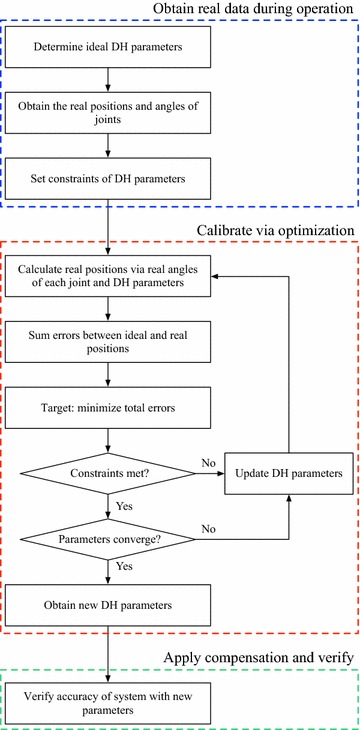
Flow chart of the optimization technique for serial manipulator robot parameter calibration and accuracy improvement

Even though the kinematics of the system can be described by the DH model, in practice, the robot arm is affected by uncertainty factors and the true DH parameters may be different from the ideal ones. If we are able to get the positions and joint angles of the robot arm as it is controlled to any position, we can calculate its DH parameters. Based on this concept, we design a calibration mechanism as the robot’s end effector. This end effector are manually placed on the calibration point to obtain its real position. We then measure the angles of joints through the camera system and encoders. A table is designed and made for robot arm calibration. In order to reduce the effects of uncertainty factors, an optimization method is applied to iteratively obtain new DH parameters. This method improves the accuracy of the robot system. The flow chart of the optimization technique for serial manipulator robot parameter calibration and accuracy improvement is shown in Fig. 8.

### Obtaining experiment data during operation

Figure 1 shows a self-built robot system. The calibration table is placed in front of the robot arms. The cavities on the table are defined as calibration points. Before the experiment, we have to get the positions of the cavities using computer-aided design. However, because there is error caused by tolerance and assembly error for the calibration table, we used the Vicon camera system to capture the positions of the cavities. When the experiment starts, the robot arm is moved to the calibration points and the angles of every joint are recorded by encoders. The real positions and joint angles are thus obtained.

**Fig. 9:**
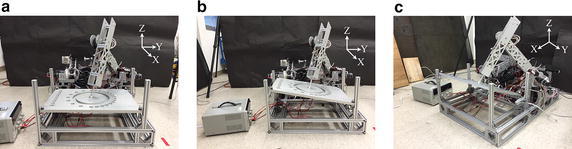
Photograph of calibration table in **a** normal, **b** roll, and **c** pitch direction

The calibration table can be directly fixed to the base of the dual-arm system, as shown in Fig. 9a. It can rotate in roll and pitch directions, as shown in Fig. 9b, c. Rotation in the roll direction is under 20°, because this is the maximum rotation angle of the ball joint. Rotation in the pitch direction is more than 20° because the ball joint can rotate 360 degrees in this direction. In this paper, the experiment was conducted at 11.7° in the roll direction and 11.8° in the pitch direction.

### Calibration via optimization

#### Optimization formulation

The optimization procedure is shown in Fig. 8. The real positions and angles are known and the ideal positions can be calculated using forward kinematics. The differences between the real and ideal positions are positioning errors of the robot arm and the sum of error at each position is considered as a objective function. Either absolute deviation or squares of the deviations can be used. In this work we use absolute value ensure the scale of the objective function matches its physical meaning in our experiments. New DH transformation matrix is calculated by changing the DH parameters. Using the new DH transformation matrix, we calculate the ideal position of the real angle through forward kinematic. The error between real and ideal position is then minimized in our optimization formulation. The function of the optimization method is shown in Eq. (4).

4 $$\begin{aligned} \begin{aligned} \min _\mathbf{DH'}~~&E = \sum ^k_{j=1}(|X_j-X'_j(\mathbf{DH'})|)\\ \text{ with } \text{ respect } \text{ to }~~~&\delta d_i, \delta \theta _i, \delta L_i, \delta \alpha _i\\ \text{ subject } \text{ to }~~~&|\delta d_i|\le e_d\\&|\delta \theta _i|\le e_\theta \\&|\delta L_i|\le e_L\\&|\delta \alpha _i|\le e_\alpha \\&E\le ka\\ \text{ where }~~~&i = 1, 2,\dots ,N\\&j=1,2,\dots ,k\\&\mathbf{DH'}=\begin{bmatrix}{} \mathbf{d'},{\varvec{\theta }'},\mathbf{L'},{\varvec{\alpha }'}\end{bmatrix}_{N\times 4}\\&\mathbf{d'} = \begin{bmatrix} d_1+\delta d_1,&d_2+\delta d_2,&\dots ,&d_N+\delta d_N\end{bmatrix}^T\\&{\varvec{\theta }'} = \begin{bmatrix}\theta _1+\delta \theta _1 ,&\theta _2+\delta \theta _2 ,&\dots ,&\theta _N+\delta \theta _N\end{bmatrix}^T\\&\mathbf{L'} = \begin{bmatrix}L_1+\delta L_1 ,&L_2+\delta L_2 ,&\dots ,&L_N+\delta L_N\end{bmatrix}^T\\&{\varvec{\alpha }'} = \begin{bmatrix}\alpha _1+\delta \alpha _1 ,&\alpha _2+\delta \alpha _2 ,&\dots ,&\alpha _N+\delta \alpha _N\end{bmatrix}^T \end{aligned} \end{aligned}$$

where *E* is the objective function of the optimization method and the summation of errors between all the ideal positions and real positions. $X_j$ and $X'_j$ are the real and ideal positions, respectively. *i* and *j* are the numbers of each joint and calibration point, respectively. *N* and *k* are the sums of joint and calibration points, respectively. $\mathbf{DH'}$ is the new DH parameters optimized in the calibration process. $d_i$, $L_i$, and $\alpha _i$ are the initial DH parameters. $\theta _i$ is the joint angle of each calibration point. The design variables $\delta d_i$, $\delta \theta _i$, $\delta L_i$, and $\delta \alpha _i$ are the deviations of DH parameters and $e_d=0.005$ m, $e_\theta$ = $2^{\circ }$, $e_L=0.005$ m, and $e_\alpha$ = $2^{\circ }$ are constraints of these parameter deviations, respectively. Finally, *a* is the average accuracy of calibration points. This research uses the MATLAB function fmincon to determine the optimized DH parameters with constraints.

## Results

A table was designed for calibrating the robot arm, and new DH parameters were obtained using the optimization process. The original DH parameters of the robot system were replaced by the new ones. A few positions were chosen to verify the accuracy of the system with new parameters. The robot arm was controlled to move to the chosen positions and the real positions were captured by the Vicon camera system. The errors were determined by comparing the ideal and real positions. The positions used to verify the robot system’s accuracy are different from the calibration points. The results are shown in Table 4 and the DH parameters are listed in Table 5.

**Table 4:** Comparison of ideal and real positions with a 95 % confidence interval (unit: mm)

Verification point	Initial error	Normal	Roll	Pitch
Point 1 (355, −275,50)	26.234 ± 0.021	15.849 ± 0.049	18.793 ± 0.034	22.375 ± 0.023
Point 2 (355, −135,50)	26.522 ± 0.039	17.444 ± 0.065	15.677 ± 0.140	20.094 ± 0.069
Point 3 (495, −275,50)	28.178 ± 0.032	17.122 ± 0.027	26.898 ± 0.038	27.686 ± 0.028
Point 4 (355, −415,50)	27.651 ± 0.075	16.329 ± 0.030	21.531 ± 0.029	23.417 ± 0.028
Point 5 (215, −275,50)	25.949 ± 0.030	16.230 ± 0.132	12.514 ± 0.025	16.230 ± 0.070
Point 6 (355, −275,150)	26.568 ± 0.038	15.643 ± 0.063	19.179 ± 0.049	23.440 ± 0.024
Average error	26.850	16.436	19.099	22.207
Improvement		38.789 %	28.868 %	17.292 %

**Table 5:** Comparison of DH parameters

i	Model	$d_i$ (m)	$\theta _i$	$L_i$ (m)	$\alpha _i$
1	Original	0.076	$\theta _1$	0	90°
	Normal	0.0755	−0.6036° + $\theta _1$	0.0003	90.0783°
	Roll	0.0740	−0.6093° + $\theta _1$	0.0020	90.0783°
	Pitch	0.0780	−0.7509° + $\theta _1$	0.0020	88.0000°
2	Original	0	90° + $\theta _2$	0.4	0°
	Normal	−0.0004	90.0704° + $\theta _2$	0.4002	−1.0250°
	Roll	0.0003	91.4311° + $\theta _2$	0.4020	−1.0250°
	Pitch	−0.0009	90.5759° + $\theta _2$	0.4020	1.8774°
3	Original	−0.012	$\theta _3$	0	90°
	Normal	−0.0124	−0.1320° + $\theta _3$	−0.0001	89.4740°
	Roll	−0.0117	−0.3118° + $\theta _3$	0.0004	89.4740°
	Pitch	−0.0127	0.4572° + $\theta _3$	−0.0002	88.0000°
4	Original	0.3543	0°	0	90°
	Normal	0.3545	0°	−0.0001	90°
	Roll	0.3563	0°	0.0004	90°
	Pitch	0.3523	0°	−0.0002	90°

**Fig. 10:**
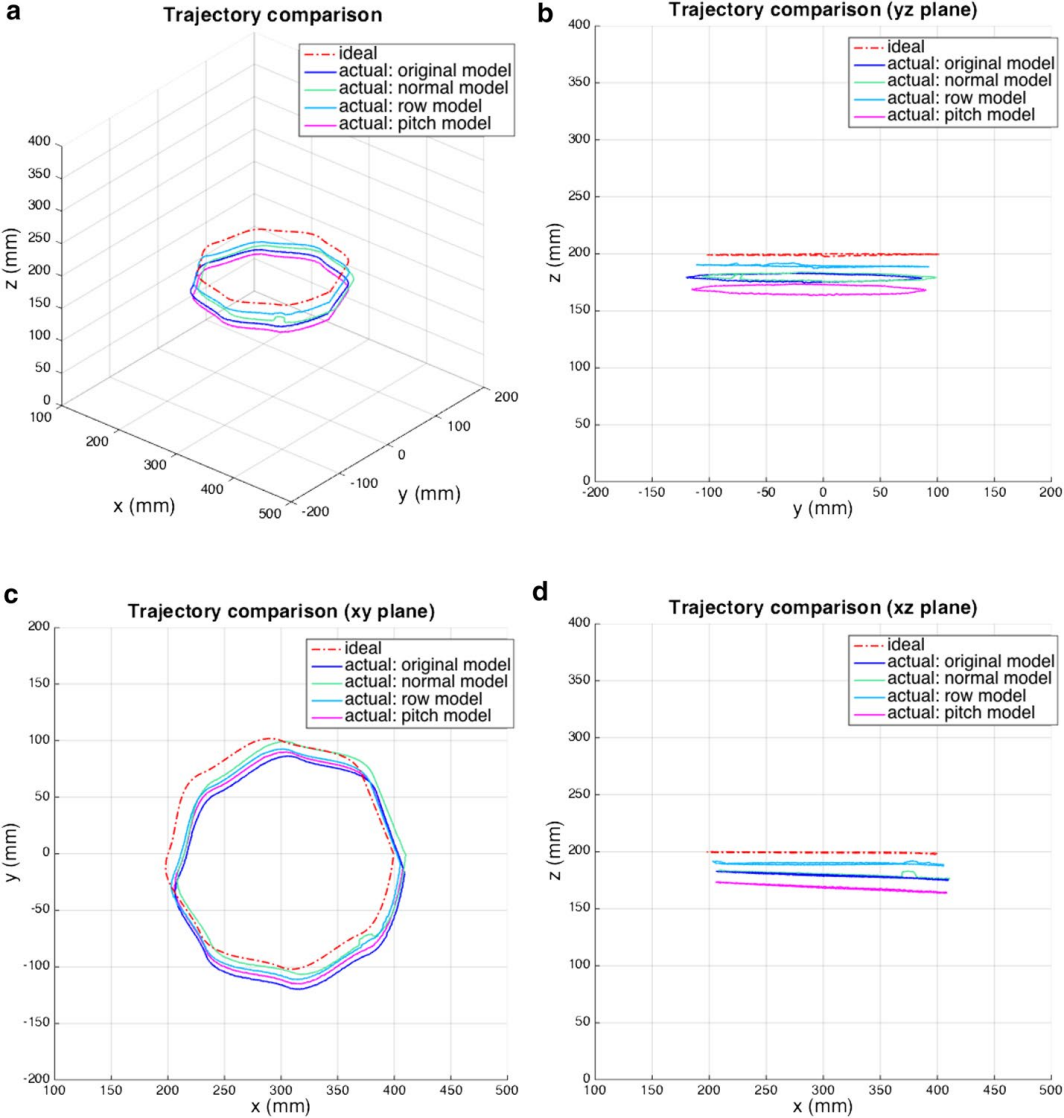
Comparison of ideal and actual trajectories with optimized DH parameters. **a** Trajectory error and views of **b** yz, **c** xy, and **d** xz planes

Table 4 shows the magnitude of improvement in the normal, roll, and pitch directions. These three directions can improve robot accuracy. Improvement in the normal direction is the best. Another comparison is shown in Table 6 and Fig.  10. We used a trajectory that is different from the verification trajectory which composed of points 1–6. The normal and roll directions still improve accuracy from 32.1 % to as much as 53.0 %, but the pitch direction has no effect. The cause of this difference is backlash. We found that the influence of motor backlash is excessively large with our robot’s structure operating pitch calibration. A different structure of the robot should be adjusted by the other direction.

**Table 6:** Comparison of ideal and real trajectory with a 95 % confidence interval (unit: mm)

Model	Center position	Offset of center	Error^a^	Improvement^b^
Ideal	(300, 0, 200)			
Original	(330.12, −17.79, 178.36)	40.75 ± 0.045	36.43 ± 0.032	
Normal	(325.04, −4.28, 179.30)	32.31 ± 0.038	24.72 ± 0.102	(32.1 ± 0.08) %
Roll	(314.35, −10.13, 189.41)	20.13 ± 0.032	17.12 ± 0.027	(53.0 ± 0.11) %
Pitch	(325.18, −13.30, 167.88)	42.37 ± 0.036	35.41 ± 0.031	(2.8 ± 0.03) %

^a^The error is measured on the trajectory compared with the ideal trajectory

^b^The improvement is compared with the original uncalibrated trajectory

## Conclusions

Robot accuracy was improved in this research. A calibration mechanism and procedure were designed for calibrating manipulators. The robot arm is moved to calibration points and its positions and angles are recorded. The errors can be obtained by comparing the ideal and real positions. Then, new DH parameters are iteratively obtained using an optimization method. To verify the results, the robot arm was moved to specific positions and its motion was captured by a camera system to determine improvement in accuracy. Robot initial average error is 26.850 mm and the average error(improvement) after calibration are 16.436(38.789 %), 19.099(28.866 %), and 22.207(17.292 %) via optimization in normal, roll, and pitch direction, respectively. The result shows that robot accuracy was enhanced at any workspace which can be chosen by user. If the error of the calibration table is small enough to be ignored, this calibration process can be conducted without a camera system. Many unknown factors affect robot errors. The robot system was very complicated and thus difficult to analyze. Although robot accuracy was improved, only geometric errors were considered. In the future, more uncertainty factors will be studied to make the robot arm more accurate.

## Appendix

### Denavit–Hartenberg transformation matrix

The DH transformation matrix is commonly used to transform coordinates from one frame to another. The convention of affixing frames was given in “Frame of the robot arm” section. In this subsection, we will construct a transformation matrix from frame $i-1$ to frame *i*. There are four steps:

- Step 1. Frame $i-1$ is translated along $z_{i-1}$ by a distance $d_i$, and its origin $O_{i-1}$ coincides with $H_{i-1}$. The transformation matrix of this step is:
  5 $$\begin{aligned} T(z,d) = \begin{bmatrix}{\begin{matrix} 1&{}0&{}0&{}0 \\ 0&{}1&{}0&{}0 \\ 0&{}0&{}1&{}d_i \\ 0&{}0&{}0&{}1 \\ \end{matrix}}\end{bmatrix} \end{aligned}$$
- Step 2. Frame $i-1$ is rotated about $z_{i-1}$ by angle $\theta _i$, and the $x_{i-1}$ axis will coincide with $x_i$. The transformation matrix of this step is:
  6 $$\begin{aligned} T(z,\theta ) = \begin{bmatrix}{\begin{matrix} \cos \theta _i&{}-\sin \theta _i&{}0&{}0 \\ \sin \theta _i&{}\cos \theta _i&{}0&{}0 \\ 0&{}0&{}1&{}0 \\ 0&{}0&{}0&{}1 \\ \end{matrix}}\end{bmatrix} \end{aligned}$$
- Step 3. Frame $i-1$ is translated along $x_i$ by a distance $L_i$, and then origin $O_{i-1}$ will coincide with $O_i$. The corresponding transformation matrix is:
  7 $$\begin{aligned} T(x,L) = \begin{bmatrix}{\begin{matrix} 1&{}0&{}0&{}L_i \\ 0&{}1&{}0&{}0 \\ 0&{}0&{}1&{}0 \\ 0&{}0&{}0&{}1 \\ \end{matrix}}\end{bmatrix} \end{aligned}$$
- Step 4. Frame $i-1$ is rotated about $z_{i-1}$ by an angle $\alpha _i$, and then frame $i-1$ coincide with frame *i* completely. The transformation matrix is:
  8 $$\begin{aligned} T(x,\alpha ) = \begin{bmatrix}{\begin{matrix} 1&{}0&{}0&{}0 \\ 0&{}\cos \alpha _i&{}-\sin \alpha _i&{}0 \\ 0&{}\sin \alpha _i&{}\cos \alpha _i&{}0 \\ 0&{}0&{}0&{}1 \\ \end{matrix}}\end{bmatrix} \end{aligned}$$

Using steps 1 to 4, the four subcomponents of the transformation matrix from frame $i-1$ to frame *i* are obtained. Post-multiplying each matrix yields:

9 $$\begin{aligned} ^{i-1}A_i = T(z,d)T(z,\theta )T(x,L)T(x,\alpha ) \end{aligned}$$

This equation can be expanded to:

10 $$\begin{aligned} ^{i-1}A_i = \begin{bmatrix}{\begin{matrix} \cos \theta _i&{}\quad {-}\cos \alpha _i \sin \theta _i&{}\quad \sin \alpha _i \sin \theta _i&{}\quad L_i \cos \theta _i \\ \sin \theta _i&{}\quad \cos \alpha _i \cos \theta _i&{}\quad {-}\sin \alpha _i \cos \theta _i&{}\quad L_i \sin \theta _i \\ 0&{}\quad \sin \alpha _i&{}\quad \cos \alpha _i&{}\quad d_i \\ 0&{}\quad 0&{}\quad 0&{}\quad 1 \\ \end{matrix}}\end{bmatrix} \end{aligned}$$

This is called the DH transformation matrix. The superscript i−1 and the subscript *i* represent the transformation from frame $i-1$ to frame *i*, respectively.

### Definition of DH parameters

The DH transformation matrix includes four parameters, namely $d_i$, $\theta _i$, $L_i$, $\alpha _i$. The convention of the parameters is described as follows.

1. $d_i$: the distance of the line that is common perpendicular to $x_{i-1}$ and $x_i$. $d_i = \overline{H_{i-1}O_{i-1}}$. If $d_i$ direction is along $z_{i-1}$, $d_i$ is positive and vice versa.
2. $\theta _i$: the angle between $x_{i-1}$ and $x_i$.
3. $L_i$: the offset between two neighboring joints and the line that is common perpendicular to $z_{i-1}$ and $z_i$.
4. $\alpha _i$: the angle between $z_{i-1}$ and $z_i$.

For a serial robot arm only with only rotational joints, these four parameters are sufficient to describe its kinematics.

### Forward kinematic method

From the obtained joint angles, the position of the system can be calculated using forward kinematics, as mentioned in “The modeling and experiment setup of a 3-DOF serial robot” section.

11 $$\begin{aligned} ^{0}A_1= \begin{bmatrix}{\begin{matrix} \cos \theta _1&{}\quad 0&{}\quad \sin \theta _1&{}\quad 0\\ \sin \theta _1&{}\quad 0&{}\quad -\cos \theta _1&{}\quad 0\\ 0&{}\quad 1&{}\quad 0&{}\quad 0.076\\ 0&{}\quad 0&{}\quad 0&{}\quad 1 \\ \end{matrix}}\end{bmatrix} \end{aligned}$$

12 $$\begin{aligned} ^{1}A_2= \begin{bmatrix}{\begin{matrix} \cos\,(90^\circ +\theta _2)&{}\quad -\sin\,(90^\circ +\theta _2)&{}\quad 0&{}\quad 0.4 \cos\,(90^\circ +\theta _2)\\ \sin\,(90^\circ +\theta _2)&{}\quad \cos\,(90^\circ +\theta _2)&{}\quad 0&{}\quad 0.4 \sin\,(90^\circ +\theta _2)\\ 0&{}\quad 0&{}\quad 1&{}\quad 0\\ 0&{}\quad 0&{}\quad 0&{}\quad 1 \\ \end{matrix}}\end{bmatrix} \end{aligned}$$

13 $$\begin{aligned} ^{2}A_3= \begin{bmatrix}{\begin{matrix} \cos \theta _3&{}\quad 0&{}\quad \sin \theta _3&{}\quad 0\\ \sin \theta _3&{}\quad 0&{}\quad -\cos \theta _3&{}\quad 0\\ 0&{}\quad 1&{}\quad 0&{}\quad -0.012\\ 0&{}\quad 0&{}\quad 0&{}\quad 1 \\ \end{matrix}}\end{bmatrix} \end{aligned}$$

14 $$\begin{aligned} ^{3}A_4= \begin{bmatrix}{\begin{matrix} 1&{}\quad 0&{}\quad 0&{}\quad 0\\ 0&{}\quad 0&{}\quad -1&{}\quad 0\\ 0&{}\quad 1&{}\quad 0&{}\quad 0.3543\\ 0&{}\quad 0&{}\quad 0&{}\quad 1 \\ \end{matrix}}\end{bmatrix} \end{aligned}$$

15 $$\begin{aligned} ^0A_4 = {^0A}_1{^1A_2}{^2A_3}{^3A_4} \end{aligned}$$

16 $$\begin{aligned} \begin{bmatrix} q_x \\ q_y \\ q_z\\ 1\\ \end{bmatrix} ={^0A_4} \begin{bmatrix} 0 \\ 0 \\ 0\\ 1\\ \end{bmatrix} \end{aligned}$$
